# Street Troughs and Glanders

**Published:** 1892-08-13

**Authors:** 


### STREET TROUGHS AND GLANDERS.

It is unpleasant to think that the numerous water troughs placed in the public thoroughfares, which are, undoubtedly, a great boon to animals, should not, as was hoped, have proved an unmixed blessing. There seems no reason to doubt that the spread of glanders in London is due to these very troughs, yet the fault does not lie in their use, but in their abuse; and anyone who knowingly waters at them a horse suffering from this disease should not escape unpunished.

